# Aesthetic Rehabilitation of a Patient with Bruxism Using Ceramic Veneers and Overlays Combined with Four-Point Monolithic Zirconia Crowns for Occlusal Stabilization: A 4-Year Follow-Up

**DOI:** 10.1155/2019/1640563

**Published:** 2019-08-20

**Authors:** André Moreira, Filipe Freitas, Duarte Marques, João Caramês

**Affiliations:** ^1^Private practice, Implantology Institute, Lisboa, Portugal; ^2^Department of Oral Surgery and Oral Medicine, Faculdade de Medicina Dentária da Universidade de Lisboa, Lisbon, Portugal; ^3^Department of Morfo-Functional Sciences, Faculdade de Medicina Dentária da Universidade de Lisboa, Lisbon, Portugal; ^4^Department of Oral Surgery and Implant Dentistry, Faculdade de Medicina Dentária da Universidade de Lisboa, Lisbon, Portugal

## Abstract

Management of severe worn dentition in patients with bruxism is challenging as a result of the loss of tooth structure and occlusal vertical dimension, temporomandibular implications, tooth hypersensitivity, and masticatory or aesthetic impairment. This case describes the 4-year follow-up clinical evaluation of a full mouth tooth-supported rehabilitation made on a 66-year-old man with bruxism and tooth wear, with aesthetic complaints and compromised masticatory function. The prosthetic treatment was planned with a digital smile design and a mock-up technique for an aesthetic and minimally invasive approach using lithium disilicate pressed and layered veneers on anterior teeth, posterior CAD/CAM lithium disilicate overlays with facial coverage, and CAD/CAM monolithic zirconia crowns with facial feldspathic ceramic on maxillary and mandibular canines and first molars in order to ensure the occlusal stability at the increased occlusal vertical dimension. After 4 years of function, no complications were registered. The choice of an appropriate material for the rehabilitation of these patients is essential to improve treatment prognosis and should be guided by mechanical and aesthetical properties. The use of four-point occlusal stabilization with CAD/CAM high strength monolithic zirconia crowns combined with ceramic veneers and overlays appears to be a reliable treatment option that enhances aesthetics and minimizes the occurrence of ceramic fractures, ensuring the treatment prognosis and predictability.

## 1. Introduction

According to the American Academy of Sleep Medicine, bruxism can be defined as the repetitive muscle activity of the jaw characterized by clenching or grinding of teeth and/or bracing or thrusting of the mandible [1]. Although the exact etiology of bruxism is still uncertain and probably multifactorial, the consequences are varied and include temporomandibular disorders, headaches, tooth wear or fracture, implant and restoration failure, tooth hypermobility, periodontal breakdown, occlusal dimpling, exostosis, muscle enlargement, and loss of occlusal vertical dimension. Bruxism is one of the most frequent causes for occlusal tooth wear, with the loss of tooth structure caused by mechanical wear between maxillary and mandibular tooth surfaces [2]. Loss of dental tissue can also result in sensitivity, pulp necrosis, and pain [3].

The prosthodontic rehabilitation of patients with bruxism must take into account the patient's needs and available materials. An appropriate material is essential to improve treatment prognosis and should combine mechanical properties and aesthetics.

The restorative approaches for patients with bruxism and worn dentition may include direct or indirect restorations such as direct resin composites or metal, gold, and ceramic and laboratory composites to make onlays or crowns, alone or in combination [4]. In cases of severely worn dentition, the traditional prosthetic treatment would include tooth preparation for complete crowns and an increase on the occlusal vertical dimension. In these cases, porcelain-fused-to-metal crowns were the traditional prosthetic option. With the introduction of new materials, zirconia has been shown to be a viable alternative to metal [5]. A systematic review about the clinical success of zirconia-based crowns suggests that the success rate of tooth-supported and implant-supported zirconia crowns is adequate, similar, and comparable to that of conventional porcelain-fused-to-metal crowns [6]. Despite the acceptable clinical results of bilayered porcelain-zirconia rehabilitations, the occurrence of ceramic chipping and fractures has been reported as a frequent complication [7].

In order to minimize ceramic fracture and chipping events, the introduction of zirconia computer-aided design and computer-aided manufacturing (CAD/CAM) has provided a new way of producing fixed restorations. A monolithic zirconia treatment option minimizes fracture events and improves structural mechanical properties [8]. Monolithic zirconia with minimal porcelain veneering limited to facial surface presents lower technical complications [9].

On the other hand, to avoid potentially harmful consequences to the teeth, non- or minimally invasive treatment options have emerged. With recent advances in dental adhesion, direct or conservative indirect restorations have become a treatment option. For many decades, ceramics have been used for anterior restorations because of their excellent aesthetic qualities and superior biocompatibility [10]. However, ceramics were brittle, required careful polishing techniques, and were abrasive to the opposite dentition [11]. Some authors suggested that placement of ceramics over the occlusal surfaces can lead to wear of the opposing dentition [12]. The use of traditional ceramic restorations on opposing occlusal surfaces is not a commonly used treatment option.

Based on this, the proposed case report describes a conservative full-mouth treatment modality of worn dentition on a patient with bruxism, using porcelain veneers and overlays combined with monolithic zirconia crowns for occlusal stabilization.

## 2. Case Report

**Figure 1:**
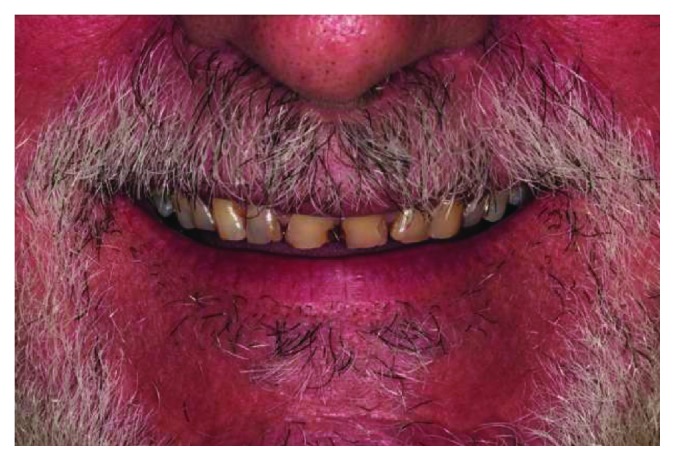
Smile (frontal view).

**Figure 2:**
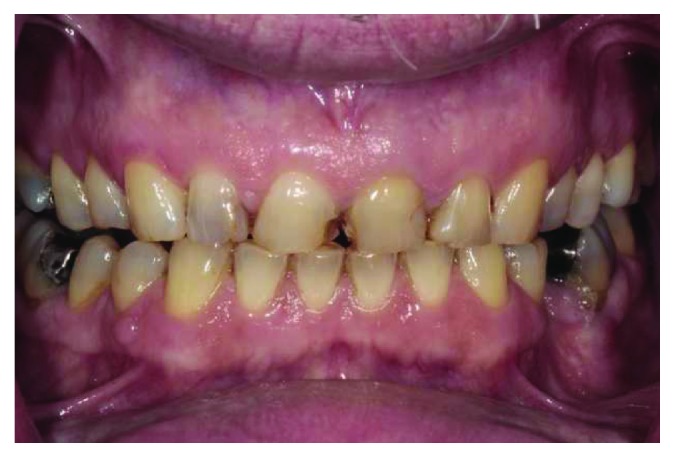
Intraoral frontal view.

**Figure 3:**
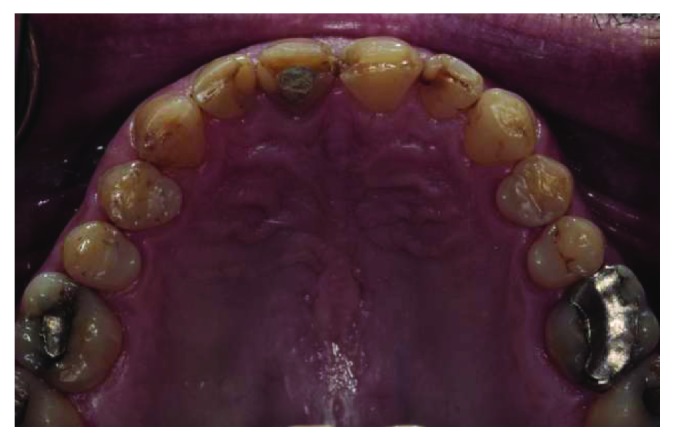
Maxillary occlusal view.

**Figure 4:**
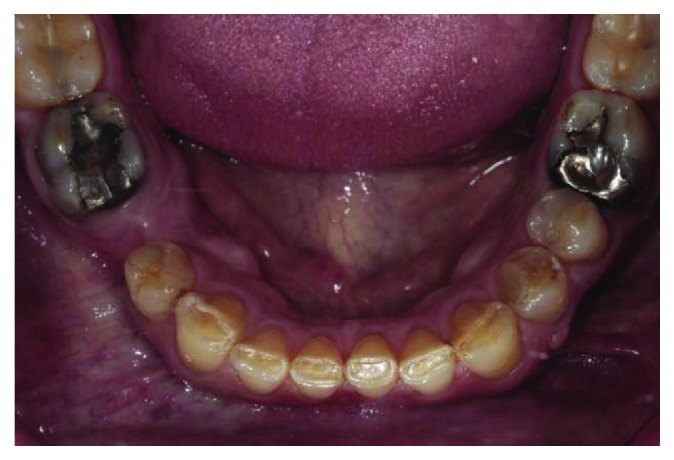
Mandibular occlusal view.

A 66-year-old man reported to the appointment with complaints related to impaired aesthetics. The intraoral clinical examination revealed the presence of worn maxillary and mandibular dentition, with dentinal craters and sharp edges on the enamel of remaining teeth (Figures 1–4).

**Figure 5:**
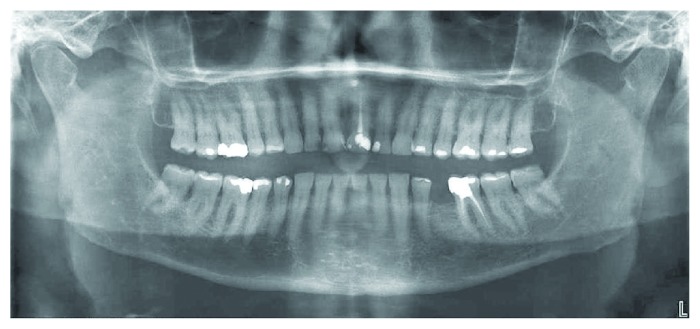
Initial panoramic view.

Upon extraoral examination, the patient showed bilateral hypertrophy of the masticatory muscles. The radiographic examination revealed the absence of tooth number 20. Teeth number 9 and 19 had previous endodontic treatment and direct composite restorations (Figure 5). Both posterior maxillary and mandibular dentition displayed worn occlusal/incisal surfaces. No anterior or canine guidance for eccentric jaw movements was present. The magnitude of occlusal vertical dimension loss was achieved using the interocclusal rest space with the jaw in rest position that was found to be around 6 mm, greater than the normal value (2 to 4 mm).

The treatment options were explained and a conservative treatment modality was adopted, which included the preparation of maxillary and mandibular canines and first molars for monolithic zirconia crowns in order to obtain four-point occlusal stability on the increased vertical dimension, that would allow to rehabilitate the anterior teeth with porcelain veneers and the remaining posterior teeth with ceramic overlays with facial coverage. In order to improve aesthetics, these monolithic zirconia crowns were veneered with porcelain in nonfunctional facial areas. A dental implant was proposed on the region of tooth number 20, but the patient decided to place a fixed bridge.

An informed consent was obtained from the patient. After facial and smile analysis, the photographic sequences were obtained and intraoral impressions were taken with irreversible hydrocolloid (Orthoprint, Zhermack). The digital planning using a digital smile design was complemented with a diagnostic wax-up that was produced on study casts and a direct mock-up with bis-acrylic composite (Protemp Plus, 3M ESPE).

All changes needed were done on the mock-up, and a silicone guide was obtained. Following this, the canines and first molars of both arches were prepared for full crowns. A medium grit diamond bur with rounded edge was used to ensure a minimum axial wall thickness for zirconia of about 1.0 mm to 1.5 mm. At gingival margin, a continuous circumferential chamfer with at least 0.5 mm reduction was made. A minimum of 1.5 to 2 mm incisal/occlusal reduction was ensured, approximately. The vertical and horizontal preparations were performed in order to obtain an angle of approximately 6 to 10 degrees between them. All edges and angles were rounded.

The anterior maxillary and mandibular teeth were minimally prepared for veneers, ensuring a minimum restoration thickness on the cervical and labial area of about 0.5 mm and 0.7 mm on the incisal edge. All other teeth were only softened from the sharp edges of the enamel.

Then, the retraction cords were applied (double retraction cord technique, #000 and #0 Ultrapak, Ultradent) and elastomeric single step impressions were made with putty and low consistency polyvinylsiloxane impression materials (Affinis, Coltene) to obtain the definitive casts. Maxillomandibular records (facebow) with the increased occlusal vertical dimension were obtained, and the master casts were mounted on a semiadjustable articulator. After tooth preparations, provisionals on the anterior teeth and first molars were placed and cemented with noneugenol temporary dental cement (TempBond NE, Kerr).

Digital technologies were then included in the workflow with the laboratory scanning of the master casts and CAD/CAM manufacturing software, along with computer-controlled machinery (Zirkonzahn).

The casts and the wax-up were scanned into the computer-aided design software in order to produce the monolithic zirconia crowns for the canines and first molar crowns. Facial cutbacks for feldspathic ceramic were made digitally in order to improve aesthetics on these crowns. These crowns were designed in such a way so that the incisal edges of the canines were included and the veneering porcelain was applied only onto nonfunctional labial/buccal areas. The monolithic zirconia frameworks were milled using CAD/CAM software according to the manufacturer's specifications (Prettau Zirkon, Zirkonzahn).

Following framework proof and occlusal adjustments of canines and first molar upper and lower crowns, ceramic was applied on the facial surfaces of the monolithic zirconia frameworks (IPS e.max Ceram, Ivoclar Vivadent) and the feldspathic veneers for the anterior maxillary and mandibular teeth were produced (IPS e.max Press, Ivoclar Vivadent).

The canine and first molar monolithic zirconia crowns were cemented according to the manufacturer's instructions. The crowns were pretreated with aluminum oxide sandblasting (110 *μ*m; 3.5 bar), steam blasted, and dried with compressed air. After the application of the bonder, the excesses were removed by compressed air and the crowns were allowed to dry for 60 seconds. The dual-cured resin cement (RelyX Unicem, 3M ESPE) was applied, and the crowns were finally inserted. After an initial polymerization of 2 seconds of light cure, all the excesses were removed and a glycerin gel was applied before the final polymerization of 120 seconds.

The anterior upper and lower porcelain veneers were cemented with resin cement (RelyX Veneer Cement, 3M ESPE).

**Figure 6:**
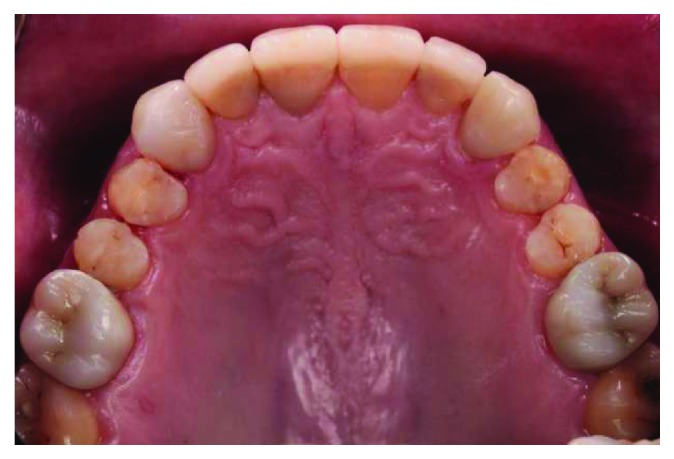
Maxillary occlusal view after initial cementation.

**Figure 7:**
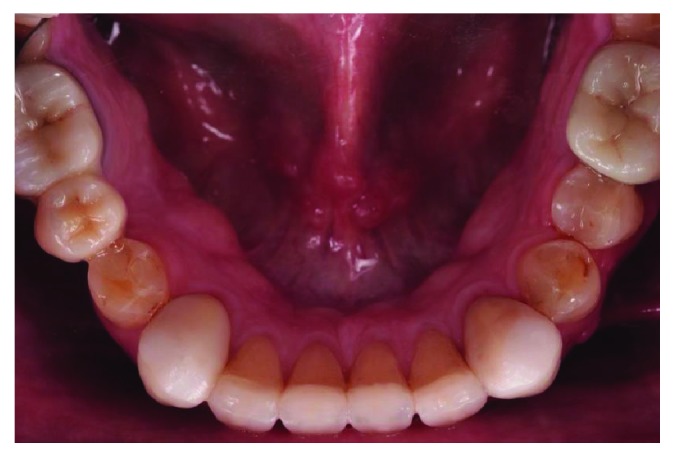
Mandibular occlusal view after initial cementation.

**Figure 8:**
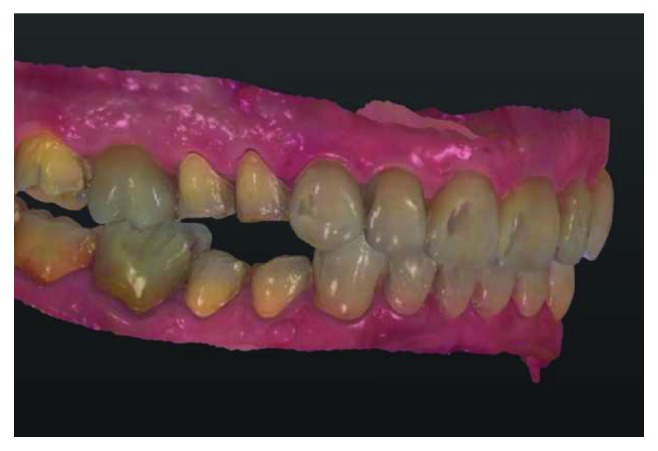
Digital scan.

**Figure 9:**
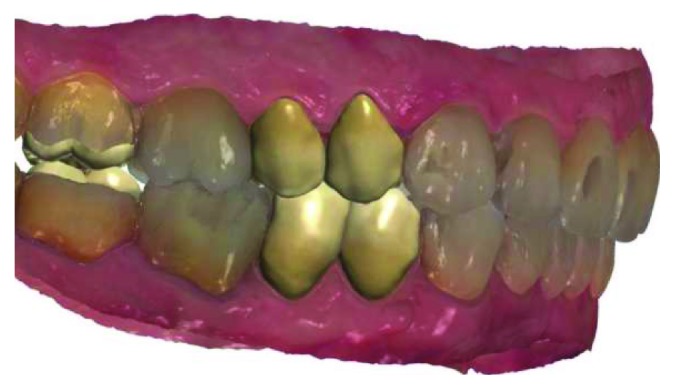
Maxillary and mandibular computer-aided design of lithium disilicate restorations.

**Figure 10:**
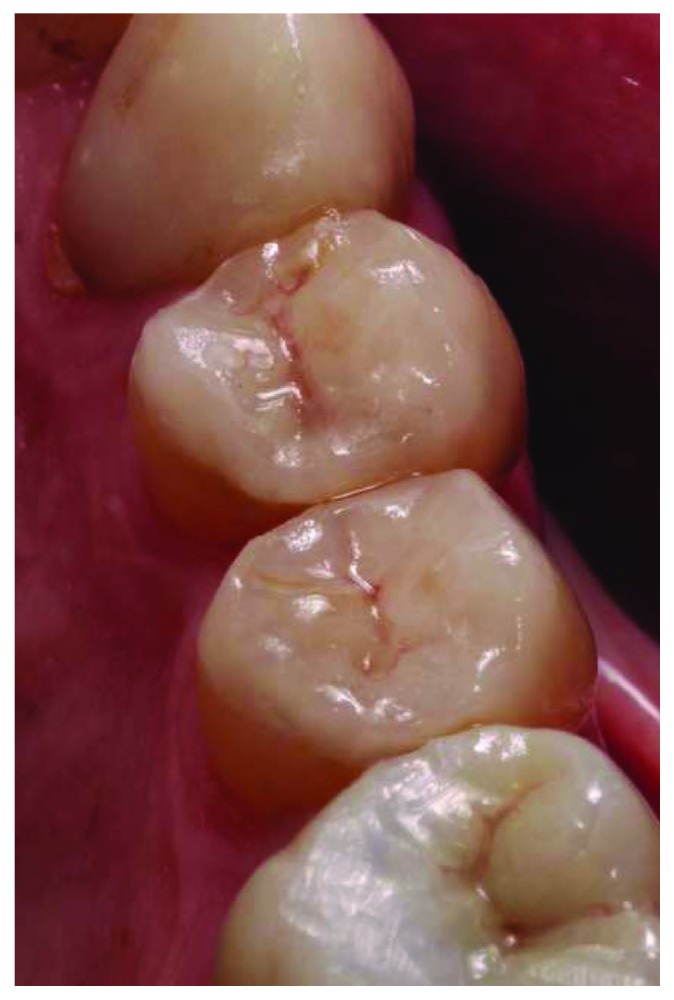
Maxillary left occlusal view after cementation of lithium disilicate restorations.

Immediately after cementation (Figures 6 and 7), a digital scan of remaining teeth of booth arches and a bite registration was obtained with an intraoral scanner (Trios, 3Shape) (Figure 8). Posterior facial and occlusal lithium disilicate glass-ceramic restorations were that obtained via CAD/CAM (IPS e.max CAD for Cerec and inLab, Ivoclar Vivadent) (Figure 9) and cemented on the same day with composite (Figure 10).

**Figure 11:**
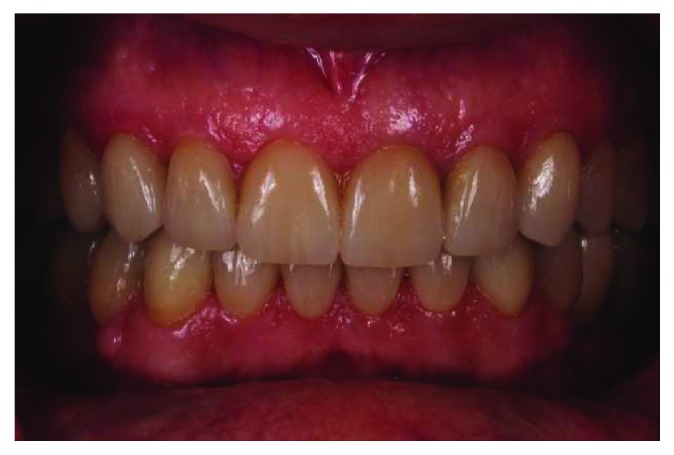
Final intraoral frontal view.

**Figure 12:**
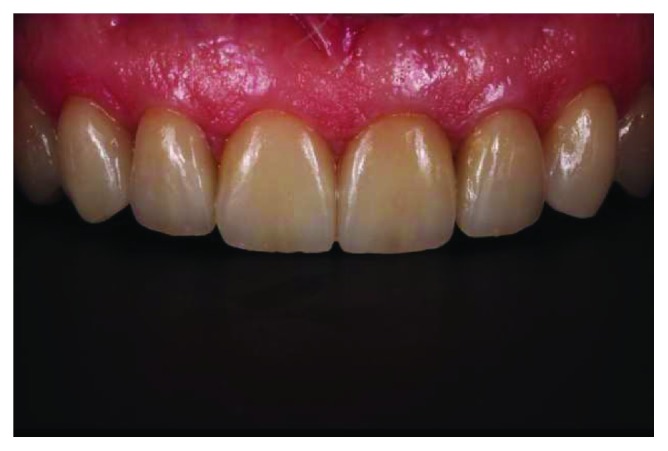
Final intraoral maxillary frontal view.

**Figure 13:**
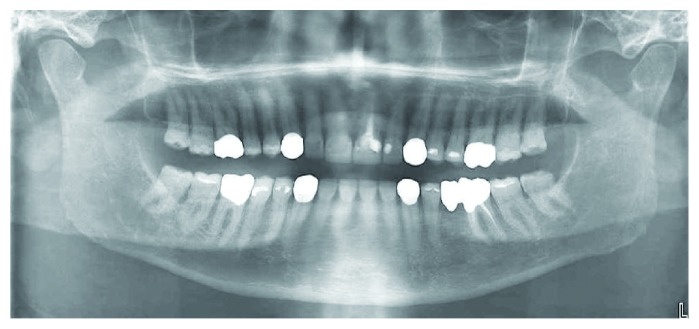
Final panoramic view.

**Figure 14:**
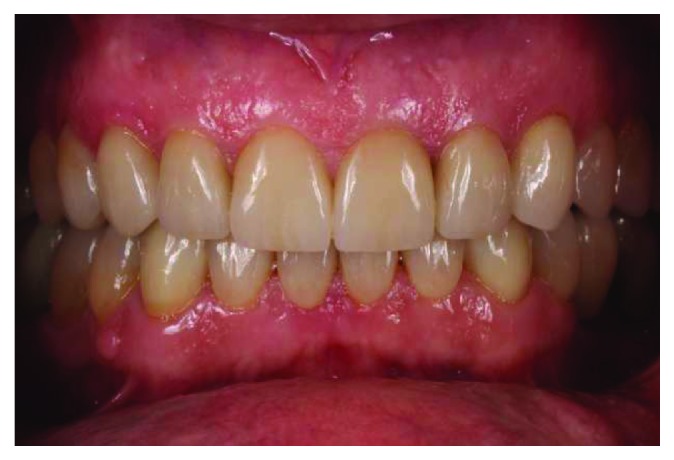
Intraoral frontal view after 48 months.

**Figure 15:**
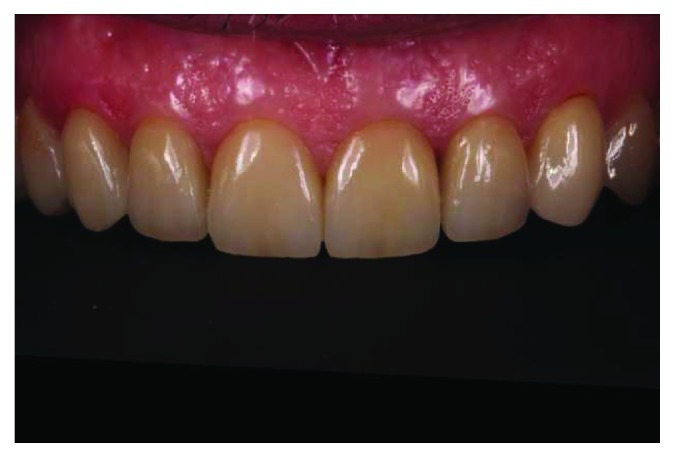
Intraoral maxillary view after 48 months.

Minor occlusal adjustments were made intraorally and polished with polishing burs. Canine guidance and anterior guidance were also verified for eccentric jaw movements with posterior disclusion. A panoramic radiograph was obtained after cementation (Figures 11–13), and oral hygiene instructions were given to the patient such as an acrylic occlusal mouthguard for nocturnal use. The patient expressed his complete satisfaction with the aesthetics and function value of the final restorations. After 4 years, no complications were found with respect to fracture or cracking of any restoration (Figures 14 and 15).

## 3. Discussion

The rehabilitation of worn dentition usually includes extensive treatment approaches. Among all the available treatment alternatives, there is a need to identify the modality that combines the best relative cost-effective with the most acceptable longevity and with the greatest benefit to the patient, for the longest period of time [13]. The right choice of the appropriate material, guided by strength and aesthetics, is crucial in determining the life of restorations and the predictability of the treatment.

The case presented showed signs of tooth wear which were attributed to bruxism. Trying to maximize aesthetics without compromising the strength and durability of the restorations and using a conservative clinical protocol, a treatment option using ceramics was proposed.

Unfortunately, for many years, some clinical evidence on ceramic failure for posterior restorations due to fracture has been reported. In addition, the use of ceramic restorations in patients with bruxism would considerably increase the risk of complications and failure [14].

This clinical report illustrates an alternative approach on the rehabilitation of a bruxer patient. Using conventional and digital techniques, maxillary and mandibular canines and first molars were restored with CAD/CAM monolithic zirconia crowns in order to ensure the occlusal stability at the increased occlusal vertical dimension. This way, the occlusal stability was ensured as well as the canine guidance.

Monolithic zirconia crowns may provide a valid treatment modality in the aesthetic zone in heavy grinders with severe tooth wear, with minor clinical complications [15]. However, the monochromic and opaque aesthetic properties of monolithic zirconia can be a limitation. The use of monolithic zirconia crowns with facial porcelain veneers can combine the mechanical strength of monolithic zirconia with the aesthetics of feldspathic ceramics, providing satisfactory clinical results with minimal biologic and mechanical complications [16]. A 1-year follow-up prospective clinical study of tooth-supported monolithic zirconia crowns made with CAD/CAM technology revealed an overall success rate of 98.5% [17].

The occlusal stabilization with high-strength monolithic zirconia crowns allowed the minimally invasive aesthetic rehabilitation of the remaining teeth, with the use of ceramic on increased vertical dimension. Thus, the anterior teeth of both arches were restored with lithium disilicate pressed and layered veneers, on a minimum tooth reduction. Lithium disilicate glass ceramics are commonly selected because of their optical properties and adhesion to the tooth structure. Furthermore, this ceramic presents slower crack propagation, better resistance, and greater biaxial strength and fracture resistance [18].

Finally, all posterior teeth (premolars and remaining molars) were restored with CAD/CAM lithium disilicate overlays with facial coverage, on a digital and noninvasive approach. Monolithic lithium disilicate overlays feature a satisfying 97.7% survival rate, on a mean follow-up of 32 months [19]. The technique allows performing restorations with a minimum thickness, minimal complications, and excellent aesthetical performance.

In order to reduce the deleterious effects of bruxism, the use of different interocclusal appliances such as soft mouthguards or hard occlusal stabilization splints has been proposed. Although there are some controversial results on the efficacy of occlusal splints in the management of bruxism, they have an important role on the prevention and limitation of dental damage caused by this disorder [20]. An acrylic occlusal mouthguard was made for nocturnal use in order to minimize the risk of fractures. The patient was advised on the importance of regular follow-ups on a 6-month recall basis. After a 4-year follow-up, no biological or prosthetic complications were registered.

## 4. Conclusion

The management of worn dentition is a major challenge for dental professionals. A correct diagnosis and a multidisciplinary treatment plan are essential to improve the treatment prognosis and patient satisfaction. The treatment decisions taken by the clinician should be based on available materials and patient demand, and the choice of an appropriate material should be guided by strength and aesthetics. Traditional options like metal-ceramic and zirconia-ceramic rehabilitations are found to be more like to chipping and fracture of ceramic.

This treatment approach appears to be a reliable treatment option with high aesthetics and strength and satisfactory clinical results minimizing technical complications. More long-term randomized control studies on the use of ceramics on grinders are necessary in order to evaluate the predictability of this kind of restorations.
